# Tracy: basecalling, alignment, assembly and deconvolution of sanger chromatogram trace files

**DOI:** 10.1186/s12864-020-6635-8

**Published:** 2020-03-14

**Authors:** Tobias Rausch, Markus Hsi-Yang Fritz, Andreas Untergasser, Vladimir Benes

**Affiliations:** 1 European Molecular Biology Laboratory (EMBL), Genomics Core Facility, Heidelberg, 69117 Germany; 2 European Molecular Biology Laboratory (EMBL), Genome Biology Unit, Heidelberg, 69117 Germany; 3 European Molecular Biology Laboratory (EMBL), Molecular Medicine Partnership Unit (MMPU), Heidelberg, 69117 Germany; 4 Center for Molecular Biology of Heidelberg University (ZMBH), Heidelberg, 69120 Germany

**Keywords:** Chromatogram, PCR, Sanger sequencing, Alignment, Variant calling

## Abstract

**Background:**

DNA sequencing is at the core of many molecular biology laboratories. Despite its long history, there is a lack of user-friendly Sanger sequencing data analysis tools that can be run interactively as a web application or at large-scale in batch from the command-line.

**Results:**

We present Tracy, an efficient and versatile command-line application that enables basecalling, alignment, assembly and deconvolution of sequencing chromatogram files. Its companion web applications make all functionality of Tracy easily accessible using standard web browser technologies and interactive graphical user interfaces. Tracy can be easily integrated in large-scale pipelines and high-throughput settings, and it uses state-of-the-art file formats such as JSON and BCF for reporting chromatogram sequencing results and variant calls. The software is open-source and freely available at https://github.com/gear-genomics/tracy, the companion web applications are hosted at https://www.gear-genomics.com.

**Conclusions:**

Tracy can be routinely applied in large-scale validation efforts conducted in clinical genomics studies as well as for high-throughput genome editing techniques that require a fast and rapid method to confirm discovered variants or engineered mutations. Molecular biologists benefit from the companion web applications that enable installation-free Sanger chromatogram analyses using intuitive, graphical user interfaces.

## Background

Sanger sequencing has a long history in molecular biology and it remains indispensable for many routine tasks like the sequencing of single genes, cloned plasmids, expression constructs or PCR products [1]. Many tools facilitate the comparison of traces with a short reference subsequence, but they lack support to align traces across entire genomes, to deconvolute mutations or to patch a reference sequence based on trace information. Automatisation of these standard tasks avoids misinterpretation of mutations and aids the researchers to focus on the critical mutations instead of inspecting hundreds of chromatogram peaks by eye. Beyond these routine chromatogram evaluation tasks that require a graphical trace analysis application, large-scale genome editing and clinical resequencing projects demand a flexible and scalable command-line application that can be integrated into automated workflows.

For large sequencing projects that aim at cataloging the human genetic variation [2] or the mutation spectrum present in diseases such as cancer [3] it is important to accurately estimate a false discovery rate of their respective call sets or to validate actionable mutations. The method of choice for this are often numerous PCR + Sanger sequencing validation experiments [4]. These validations entail the verification of a frequency-weighted subset of variants across predicted heterozygous and homozygous carriers for population studies or comparing validation results of tumor against control for cancer genomics applications. At present, these validation exercises require large-scale but often manual chromatogram trace analyses with very limited tool support. Most of the available trace analysis software aims at analyzing one trace at a time in an interactive, often proprietary and licensed trace analysis viewer that lacks support for standard file formats such as VCF/BCF [5], the predominant variant calling reporting format in NGS studies.

With the advent of genome editing tools such as CRISPR/Cas9 or TALENs there is also a growing need for rapid and easy-to-use tools to validate engineered mutations and estimate the rate of editing [6, 7]. This often demands a deconvolution of Sanger chromatogram traces into its constituting alleles, which is non-trivial for mixed chromatogram traces that involve heterozygous insertions or deletions [8]. The deconvolution of mixed chromatograms by hand is a daunting task and simple profile alignments often do not correctly resolve double mutations, where both alleles are mutated. Prior methods aiming at validating genome editing events are TIDE [7], CRISP-ID [9] or CRISPR-GA [10].

Contrary to the large-scale, automated trace analyses use case, there are plenty of tools available to visualize and interpret Sanger sequencing results using a graphical user interface, at least if commercial providers are an option. Many companies do have trace analysis software in their portfolio, including ThermoFisher and Qiagen or cloud-service providers such as Benchling (among many others). Likewise free or shareware software does exist such as GeneScreen [11], novoSNP [12] and FinchTV [13], with albeit more limited functionality. In particular, methods for deconvolution and assembly of chromatogram traces are scarce and only very few packages offer a scalable command-line application such as the Staden Package [14] or the phred/phrap/consed set of tools [15], both of these methods unfortunately still lack support for NGS-inspired formats such as VCF/BCF [5].

We propose a free and open-source method, called Tracy, a flexible and versatile method to automate Sanger trace analysis that provides a range of interactive, companion web applications as well as a scalable backend for large-scale use cases. Tracy makes use of state-of-the-art browser technologies and NGS data formats to address pressing needs for Sanger trace analyses tools that can be readily integrated in NGS workflows and genetic engineering pipelines.

## Implementation

We first describe the backend implementation of Tracy, which is a command-line application available for Linux and Mac operating systems. Afterwards, we describe the companion web applications that visualize the output of Tracy and allow interactive exploration of the results.

### Tracy command-line application

The Tracy command-line application is written in C++. The code depends on the SDSL-lite library [16] for building reference genome indices, Boost for general data structures and algorithms, HTSlib [17] for handling variant calls and BCF output, and the JSON library for modern C++ (https://nlohmann.github.io/json/). Tracy itself uses subcommands to index, basecall, align, decompose and assemble Sanger chromatogram trace files. Each subcommand is explained below.

#### Indexing

The tracy index subcommand builds an FM-Index of FASTA reference genomes and stores it to a file. These FM-Indices are required to quickly anchor chromatogram files in entire genomes for the alignment and decomposition functions of the proposed method.

#### Basecalling

Tracy can handle various trace file formats as input such as abi, ab1 and scf. Besides the trace signal for A, C, G and T, Tracy also reads the basecalling intervals that are subsequently used to generate new basecalls according to a user-defined signal-to-noise ratio as in SangerSeqR [8]. For a given basecall interval, zero to all four trace signals (A, C, G, T) can be above the user-defined peak threshold. The primary basecall is always the highest peak or ’N’ if none of the traces is above the threshold. In case of multiple traces above the peak theshold the secondary basecall reflects the IUPAC DNA code of all remaining traces, or ’N’ if all four traces are above the threshold. Tracy also supports re-estimating basecalling qualities. The output of Tracy can be in JSON, FASTA, FASTQ or TSV format. The JSON and TSV formats output the trace at every sampling position. These formats also list the basecalling positions, the primary, secondary and consensus nucleotides at each basecalling position and the estimated basecall qualities. The JSON format provides the succinct input of our trace file viewer web application, called teal (described below). The FASTA and FASTQ output formats are mere convenience methods requested by users to enable a rapid integration into existing mapping and alignment pipelines.

#### Alignment

The tracy align subcommand is used to map a chromatogram trace to a reference sequence. Notably, this reference sequence can be

- an indexed genome of several gigabases in size,
- a short sequence in FASTA format or
- a wildtype chromatogram trace

For an indexed genome the mapping happens in a classical three step process, with (1) a seeding step to anchor the trace in the reference genome using the genome’s index data structure, followed by (2) a semi-global profile-to-sequence alignment of the quality-trimmed trace to the local reference subsequence to find a reliable core alignment region, and finally (3) a full profile-to-sequence alignment of the entire trace. For short reference sequences or wildtype chromatogram files tracy directly aligns the input trace to the input sequence or wildtype chromatogram using profile-to-sequence or profile-to-profile alignments, respectively. All alignment algorithms are based on dynamic programming using data structures and algorithms derived from Alfred [18]. The final trace alignment can be produced in JSON or in ClustalW format. The ClustalW alignment format can be convenient for downstream processing tools but it obviously requires reporting a consensus nucleotide for each trace position and thus, it lacks the full trace information. This aligned trace information with all padded trace signals according to the alignment is available only in the JSON format that is also utilized by our companion web application to render a trace-to-sequence alignment, called Sage (see below).

#### Trace decomposition and variant calling

PCR amplifications do not discriminate the maternal and paternal allele present in diploid organisms, except for specialized techniques such as allele-specific PCR. Heterozygous single-nucleotide varaints can be readily identified using the secondary basecalls but heterozygous insertions or deletions introduced by gene editing techniques such as CRISPR/Cas9 cause downstream shifts in the trace signal where both alleles overlay each other with plenty of secondary basecalls. The process of disentangling these overlaying signals into two distinct alleles by means of a reference sequence is called trace decomposition. The basic idea is to thread the reference through the primary and secondary basecalls for the construction of one allele and keep the remaining basecalls as the second allele. Unlike other methods, tracy’s decomposition methods are generic and they can identify mutations independent of a pre-defined guideRNA used for genetic engineering. As for the chromatogram trace alignment, trace decomposition works for an indexed genome of arbitrary size, a short FASTA sequence or a wildtype chromatogram as reference input. Once the two alleles have been identified, variant calling is performed by simply aligning the different alleles to the reference sequence with a separate genotyping step to assign variant qualities and zygosity. For pre-built genome indices that are available in Ensembl tracy further queries the Ensembl API for known variants and annotates all catalogued variants with their rs identifiers. Based on the heterozygous variant calls and the original chromatogram trace, tracy then estimates the allelic fractions of the two called alleles because in gene editing studies often only a subset of the cells might carry a mutation. Hence, the allele frequency of a heterozygous InDel might deviate from the expected 50%. Tracy estimates these allelic coefficients, reflected by the primary and secondary basecalls, by computing the optimal reconstruction of the original trace signal. The output of tracy decompose is in BCF format for the variant calls. This facilitates an easy comparison of called NGS variants with validated Sanger-sequencing derived variant calls using standard pipelines for normalizing (left-aligning) variant calls and intersecting variants. Notably, the BCF output contains the variant position, reference and alternative alleles, rs identifiers, genotypes and genotype qualities as well as the basecall position and signal position in the original trace file, which are used in our companion web application Indigo (see below) to interactively link variants to the original chromatogram trace. The method further outputs the estimated allelic fractions, the decomposition error for inferred heterozygous insertions or deletions and three alignments: (1) first allele against the reference, (2) second allele against the reference and (3) the two alleles aligned with each other.

#### Trace assembly

The last module of Tracy performs trace assembly on a set of input chromatogram trace files. This module supports two modes of operation: (1) a local reference-guided trace assembly or (2) a de novo trace assembly. For a reference-guided assembly, all traces are first aligned to the reference one-by-one using profile-to-sequence dynamic programming of the forward and reverse-complement trace profile. We then align traces progressively to a growing multiple sequence alignment (MSA), starting from the reference and then incorporating traces from highest alignment score to lowest in the best-scoring alignment direction (forward or reverse). Notably, tracy switches from an initial profile-to-sequence alignment for the first trace to a profile-to-profile dynamic programming algorithm once the second trace is aligned to the MSA of the reference and the first aligned trace. In all subsequent alignment iterations, profile-to-profile alignments are used that ignore all leading and trailing gaps in the MSA for each trace. For a de novo trace assembly, tracy employs a progressive multiple sequence alignment algorithm [19]. The first step is to compute all pairwise trace overlap alignments with affine gap penalties to build a guide tree. Traces are then progressively aligned along the guide tree using profile-to-profile alignments that ignore leading and trailing gaps. The final alignment computed at the root of the guide tree represents the MSA of all traces. The output of the assembly module consists of a standard FASTA alignment in horizontal and vertical format that uses for each alignment and trace position the consensus nucleotide or gap of the respective chromatogram trace. Such a classical MSA can be browsed and interactively explored using our web application sabre (see below). For patching reference sequences based on chromatogram traces, researchers need to be aware of secondary basecalls and the trace signal itself. To facilitate applications for this purpose (i.e., pearl described below) the assembly module also outputs a succinct JSON file that lists all padded trace signals according to the MSA.

### Companion web applications

Tracy’s front end applications are all hosted on GEAR, a web server for molecular biology applications (https://www.gear-genomics.com). Each application focuses on a single task and was designed for the interactive usage in a molecular biology lab. All applications follow the classical client-server architecture where each individual web app calls a different subcommand available in Tracy. The web applications are

- Teal: Viewing a trace file
- Sage: Aligning a trace to a reference sequence
- Indigo: Decomposing a trace, variant calling and annotation
- Pearl: Patching FASTA sequences based on a local trace assembly that optionally uses a reference sequence

**Fig. 1:**
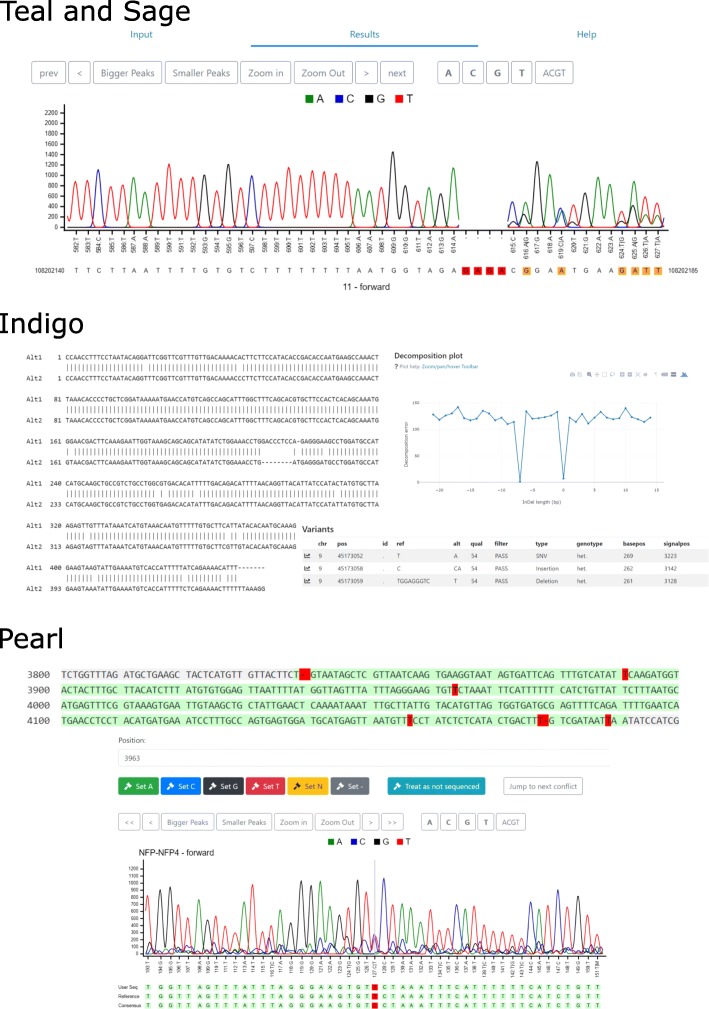
Tracy web applications. The upper panel shows the trace viewer employed by Teal and Sage. The top toolbar of the viewer provides multiple buttons to scroll along the trace, zoom and highlight the trace signals for A, C, G and T. Sage augments the basic trace viewer of Teal by the reference alignment that highlights mismatches and gaps with respect to the reference sequence. The upper panel shows a trace alignment to the region chr11:108,202,140-108,202,185 with a 4bp deletion (red) and several single-nucleotide variants with double-peaks in the trace (yellow). The middle panel (Indigo) shows the allelic decomposition of a heterozygous 7bp deletion, the textual alignment of both alleles and a subset of the called variants. The trace aligned to the reference in reverse-complement orientation but the variants are reported based on the forward strand to comply with the VCF specification. The decomposition plot shows two minima, one allele (Alt1) has no insertion or deletion (local minimum at 0bp) and the other allele (Alt2) has a 7bp deletion (local minimum at -7bp). The variant table lists all called variants with respect to the reference, their genotype and the original basecalling and signal position in the trace, which are connected via hyperlinks to the trace viewer (not shown). The lower panel (Pearl) shows the reference sequence covered by traces (green) with mismatches highlighted in red. Below is the user interface of how to patch these reference mismatches for a selected position (3963, bold and red T) with the local trace information surrounding the T mismatch. The primary basecall at this position is a C, the secondary basecall a T. The buttons in the top toolbar can be used to edit the reference sequence based on the provided trace information. Conveniently, one can directly jump from one conflict position to the next without having to scroll through all traces

Each application has its dedicated Python server running on GEAR. The basic design principle is that the client sends all input files and input parameters to the server, the server performs parameter checks and runs tracy. Once tracy completes, the server sends the results back to the client application which renders the results and allows an interactive exploration of the output. Example user interface panels of the various applications are shown in Fig. 1.

#### Teal

The trace viewer Teal has a clean graphical user interface with a single input field for uploading a trace file in scf, abi, or ab1 format. There is also an example button and a small help screen for first time users. The actual results page contains a trace viewer that allows iterating the trace, zooming, highlighting A,C,G, or T and a text field for manually editing or copy-and-pasting the basecalls. Further links to the open-source code and an email help page are provided in the GEAR header, as is the case for all other GEAR applications.

#### Sage

The trace alignment application Sage takes a chromatogram file as input together with a selected reference file which is either a pre-indexed genome available on GEAR, a single FASTA file or a wildtype chromatogram. The Sage alignment viewer follows the design of Teal, except that the trace is additionally padded for inserted or deleted nucleotides with respect to the reference. Matches are highlighted in black whereas mismatches are either highlighted in red or yellow, where red indicates a mismatch to both the primary and secondary basecall and yellow indicates a match for the secondary basecall but a mismatch for the primary basecall. The chromatogram basecalls and the matching reference subsequence are available for editing or copy-and-pasting in a text field.

#### Indigo

The application Indigo is for calling and annotating variants in chromatogram traces and for decomposing heterozygous insertions and deletions. The inputs are a chromatogram file and a reference file (genome index, short FASTA sequence, or wildtype chromatogram). The output page has multiple sections:

- A standard trace viewer
- The alignments of the two identified alleles with respect to the reference
- The alignment of the first allele to the second allele with their estimated allelic fractions
- A variant table with the reference position, basecall position and signal trace position for each variant that are connected via hyperlinks to the original trace
- A decomposition chart showing the decomposition error for each possible heterozygous InDel length

The variant table can be downloaded in CSV or BCF format. Indigo also provides a summary PDF file for download.

#### Pearl

Pearl is used to patch a reference sequence with several Sanger chromatogram trace files. If a reference sequence is provided, Pearl highlights the conflicts and mismatches between the reference sequence and the trace files. If no reference is provided, a consensus sequence is assembled and used as the reference sequence. Pearl provides a color-coded sequence overview:

- light green - consensus: all traces support the same nucleotide
- red - mismatch: traces agree on different nucleotide compared to the reference
- orange - conflict: conflict, some traces suggest other nucleotides
- green - edited: the nucleotide was entered manually by the user
- grey - no information: only reference data is available at this position

By design, Pearl focuses only on one location at a time with the position indicated below the color-coded overview. A position of interest can be selected by either clicking on a nucleotide in the overview sequence or by changing the number in the position field. Alternatively, one can also navigate the trace windows. For a selected nucleotide, the available traces can be reviewed by the user and based on this manual evaluation, the user can patch (set) the sequence to a certain nucleotide. With all mismatches and conflicts edited by the user, the sequence can be exported as a FASTA file. At any time the entire dataset, including the traces and the current patching state, can be downloaded as a JSON file to be resumed later.

### Auxiliary web applications

#### Sabre

Pearl operates one alignment position at a time. If users also wish to browse the multiple sequence alignment (MSA) of all traces in parallel they can use our MSA viewer sabre, which is a client-only web application that highlights mismatches and ambiguous DNA nucleotides in the consensus alignment.

#### Wily-DNA-Editor

**Fig. 2:**
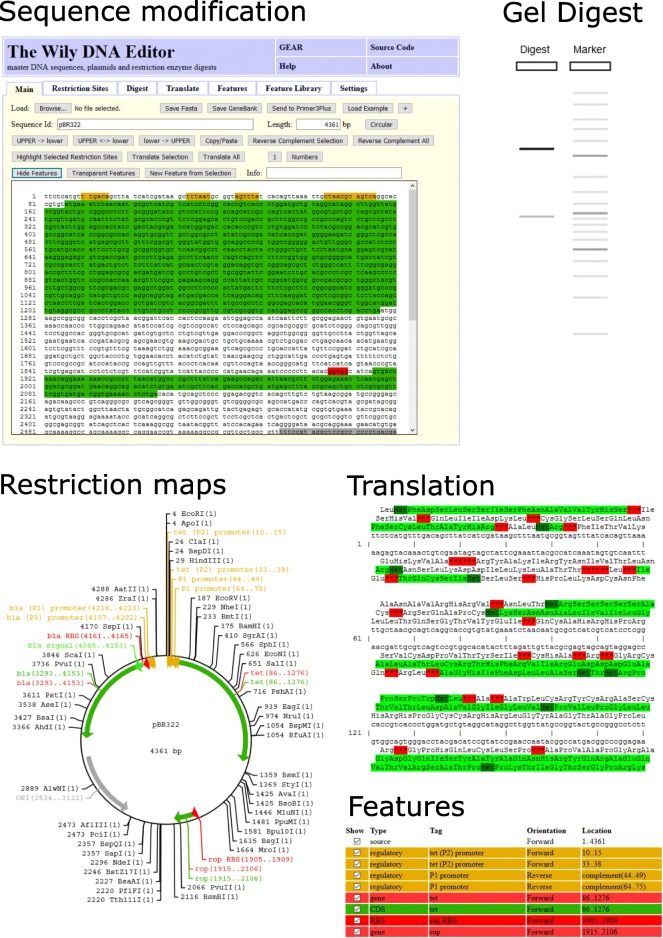
The Wily DNA Editor. In the upper left corner, an example sequence pBR322 is displayed with features highlighted in green, yellow and red as indicated in the feature table in the bottom right. Simple buttons in the top toolbar support a range of sequence manipulations such as reverse complement, upper/lower case conversion or translation to an amino acid sequence. A user-defined feature library (bottom right) can be used for sequence highlighting. The upper right shows an in-silico gel digest of pBR322 with Eco RV and Pst I including simulated band densities. Restiction site maps (bottom left) and amino-acid translations of the DNA sequence are also supported. The start codons are colored dark green, stop condons red and open reading frames in light green

Plasmid sequences from regular GenBank or FASTA files, sequences patched by Pearl or chromatogram sequences in general can be further analysed with our Wily-DNA-Editor to evaluate DNA properties and simulate cloning steps. Wily is a client-only web application that supports routine DNA sequence manipulations such as reverse complement, highlighting subsequences, upper/lower case or copy-and-paste. Open reading frames can be annotated and translated to its amino acid sequence. Cutting sites of restriction enzymes can be discovered, counted and highlighted in the sequence, displayed as a restriction site map or visualized as a virtual gel-like digest (see Fig. 2). All plots and graphs can be saved in svg format. Wily also supports a user-defined annotation library of DNA sequences to automatically scan new sequences for arbitrary DNA patterns. This can be used, for instance, to automatically annotate typical sequences for antibiotic resistance or fluorescent proteins.

## Results

As shown in Table 1, Tracy offers a very comprehensive feature set compared to other non-commercial tools. Unlike many other tools that either constitute a pure command-line tool or a standalone desktop application, Tracy is highly flexible. It can be used instantly and interactively by wet-lab researchers on the web without the need to install any tool or dependencies. For large-scale use cases in bioinformatics it however also excels by a simple to install backend available as a Docker or Singularity container, static binary or as a bioconda application. Using basic shell scripts, one can easily use the command-line application to compare hundreds of NGS variant predictions with their chromatogram validation results in a fully automated fashion. This is a unique feature that is to the best of our knowledge not readily available in other packages either because of GUI restrictions or the lack of appropriate output formats in VCF or BCF. Notably, Tracy is also one of the few methods that aligns and decomposes chromatogram traces against gigabase-sized genomes, as shown in Table 2 for CRISPR-Cas9 knockout screens from a subset of published trace files [9]. This works within seconds, which makes it completely unnecessary to extract a local reference for each and every sequenced trace file. Unlike other methods, the trace decomposition works independent of guide RNAs and it is able to discover much larger InDels up to 1000bp for a maximum of two alleles. The intutive user interface of Pearl is to the best of our knowledge unmet by other tools and its focus on patching a given sequence with several trace files at the same time greatly speeds up the semi-automated editing of FASTA sequences.

**Table 1:** Tracy feature comparison

*Core functions*	Tracy	novoSNP	GeneScreen
Basecalling	✓	✓	✓
FASTA alignment	✓	✓	✓
Wildtype chromatogram alignment	✓	✓	✓
Large genome alignment	✓	✗	✗
*Variants*			
InDel decomposition	✓	✓	✓
Variant calling	✓	✓	✓
Variant genotyping	✓	✓	✓
Variant annotation	✓	✓	✓
*Assembly*			
Trace assembly	✓	✓	✗
Patching reference sequences	✓	✓	✗
*File support*			
Trace formats (abi, ab1, scf)	✓	✓	✓
BCF/VCF support	✓	✗	✗
*GUI*			
Windows Desktop Application	✗	✓	✓
Web applications	✓	✗	✗
Command-line application	✓	✓	✗
*Distribution*			
Free and open-source for commercial and private use	✓	✗	✗
Source code distribution package	✓	✓	✗
Binary executable	✓	✓	✓
Availability of Docker/Singularity container	✓	✗	✗
Bioconda package	✓	✗	✗

**Table 2:** InDel decomposition with runtime and memory usage for mammalian-sized genomes

*Clone*	Genome	CHROM	POS	REF	ALT	runtime	memory
*Fxr1*_#1	GRCm38	3	34065038	AGAAGATAGACAGCCAGGTA	A	2 sec	1.8 GB
*Fxr1*_#2	GRCm38	3	34065050	G	GG	2 sec	1.8 GB
*MBTPS1*_#1	GRCh38	16	84101632	ACTGTT...30nuc...GAACAGCCAGGGC	A	3 sec	2.1 GB
	GRCh38	16	84101633	CTGTTG...30nuc...AACAGCCAGGGC	C	3 sec	2.1 GB
*MBTPS1*_#2	GRCh38	16	84101631	AACTGT...30nuc...GGAACAGCCAGGG	A	3 sec	2.1 GB
	GRCh38	16	84101634	TGTTGA...30nuc...ACAGCCAGGGC	T	3 sec	2.1 GB
*MBTPS1*_#3	GRCh38	16	84101631	AACTGT...30nuc...GGAACAGCCAGGGCA	A	3 sec	2.1 GB
	GRCh38	16	84101633	CTGTTG...30nuc...AACAGCCAGGGC	C	3 sec	2.1 GB

## Conclusions

Tracy is a comprehensive suite of tools for analysing chromatogram trace files. The backend complements existing trace analysis methods by means of providing a flexible and scalable tool that can be readily integrated in automated, large-scale PCR+Sanger validations of NGS variant calls or as an unsupervised verification method for genetic engineering techniques. The companion web applications were designed with the needs of a wet-lab researcher in mind and they are comparable to state-of-the-art commercial tools. All tools are readily accessible to the research community, open-source and freely available without the need to register or pay a license fee. Given the range of applications and use cases that Tracy addresses, we believe the method is a key facilitator to enable rapid trace analysis in the context of large resequencing projects where actionable variants are routinely validated prior to clinical decision making. Future development in the context of Tracy are upstream tools that automatically select primers for NGS variant calls or design primers for multiplex PCR assays. With these additions Tracy would cover the entire molecular biology workflow from primer design, PCR and Sanger sequencing to trace analysis.

## Availability and requirements

Project name: Tracy Project home page: https://github.com/gear-genomics/tracyWeb applications: https://www.gear-genomics.comOperating systems: Web applications are platform independent, the tracy command-line application is for Unix (Linux, macOS) Programming languages: C/C++, JavaScript, HTML, CSS, SVG Other requirements: No License: BSD (Tracy), GNU General Public License v3.0 (Web applications) Any restrictions to use by non-academics: No

## Data Availability

Source codes of Tracy and companion web applications are made freely available at: https://github.com/gear-genomics

## Abbreviations

API: Application programming interface
BCF: Binary variant call format, binary version of VCF
CSV: Comma-separated values, a delimited text file format
DNA: Deoxyribonucleic acid
InDel: Insertion or deletion
IUPAC DNA code: International Union of Pure and Applied Chemistry (IUPAC) notation to represent the four nucleotides
JSON: JavaScript Object Notation, a lightweight data-interchange format
MSA: Multiple sequence alignment
NGS: Next generation sequencing
PCR: Polymerase chain reaction
PDF: Portable document format
SNV: Single nucleotide variation
TSV: Tab-separated values, a delimited text file format
VCF: Variant call format
